# Computer-Aided Design and Computer-Aided Manufacturing Technology for Conducting Nasoalveolar Molding for Infants With Cleft Lip and Palate: A Scoping Review

**DOI:** 10.1177/10556656251363400

**Published:** 2025-09-11

**Authors:** Bach Kim Nguyen, Camila Caro, Kyle Stevens, Gabriella A Garisto, Yoav Finer

**Affiliations:** 1 Graduate Orthodontics, Faculty of Dentistry, 7938 University of Toronto, Toronto, Ontario, Canada; 2 Division of Orthodontics, Department of Dentistry, The Hospital for Sick Children, 7938 University of Toronto, Toronto, Ontario, Canada; 3 Division of Pediatric Dentistry, Department of Dentistry, The Hospital for Sick Children, 7938 University of Toronto, Toronto, Ontario, Canada; 4 Faculty of Dentistry and Institute of Biomedical Engineering, 7938 University of Toronto, Toronto, Ontario, Canada

**Keywords:** presurgical infant orthopedics, digital, 3D printing

## Abstract

**Objective:**

To identify, describe, and characterize computer-aided design and computer-aided manufacturing (CAD/CAM) methods for nasoalveolar molding (NAM) based on a structured search of scientific literature.

**Design:**

Scoping review was conducted following the PRISMA-ScR guidelines. Searches were done in MEDLINE, Embase, Web of Science, Cochrane Library, and Scopus. Screening and data extraction were performed.

**Patients:**

Infants with unrepaired, nonsyndromic, complete unilateral cleft lip and palate (UCLP), or bilateral cleft lip and palate (BCLP).

**Intervention:**

CAD/CAM NAM.

**Main Outcome Measures:**

Outcome measures were the digitization, virtual modeling, and manufacturing protocols.

**Results:**

Thirteen articles were included. CAD/CAM NAM involved digitizing the maxilla, designing step-by-step (stepwise) alveolar movements or expansion of plates, then manufacturing plates manually or through 3D printing. Four methods were characterized based on the virtual modeling and manufacturing techniques employed: stepwise alveolar molding and manually fabricated plates (SM_MP); stepwise alveolar molding and 3D-printed plates (SM_3P); stepwise plate expansion and 3D-printed plates (SPE_3P); and semi-automated plate expansion and 3D-printed plates (SAPE_3P). The SM_MP method was the most common, followed by the SM_3P, SPE_3P, and SAPE_3P methods. All methods were applied to treat infants with UCLP, whereas only the SM_MP and SM_3P methods were used for infants with BCLP.

**Conclusions:**

This scoping review provides an overview of 4 CAD/CAM methods for NAM. The SM_MP and SM_3P methods simulate alveolar molding; however, the SM_3P method exhibits more advanced design and manufacturing of plates. The SPE_3P and SAPE_3P methods design consecutively enlarged plates, with the latter employing a semi-automated protocol.

## Introduction

Cleft lip and palate (CLP) represents one of the most common congenital craniofacial anomalies. It occurs in approximately 1 per 700 births worldwide, with variation in prevalence occurring among different ethnic groups and geographic locations.^1–3^ Complete unilateral CLP (UCLP) is characterized by separated lip segments, a wide nose with depressed lateral alar cartilage on the cleft side, protruded greater alveolar segment, and medially collapsed lesser alveolar segment.^4,5^ Complete bilateral CLP (BCLP) presents with separated lip segments, a wide nose with a broad depressed tip, a protrusive premaxilla, and medially collapsed lateral alveolar segments.^5^

The severity of the cleft alveolar and nasal defects can be improved in infancy with presurgical infant orthopedics (PSIO) prior to primary lip and nasal surgeries. Nasoalveolar molding (NAM) developed by Grayson in 1993 is a semiactive PSIO treatment using an acrylic plate with a nasal stent for alveolar and nasal molding.^4^ The plate is secured in the infant's mouth full time by attaching orthodontic elastics around the retention arm(s) of the plate and taping them to base tapes on the infant's cheeks. In addition, lip taping can be used in conjunction with the plate. The goals of NAM are to reduce the alveolar cleft(s), align the alveolar segments, and improve nasolabial morphology.^6^ The plate can be adjusted chairside, via selective removal and addition of acrylic, to guide movements of the alveolar segments under elastic tension forces. For UCLP, the greater alveolar segment is directed medially with removal of hard acrylic at the palatal side of the greater segment, and addition of soft acrylic on the labial side of the greater segment.^7^ The position of the lesser alveolar segment can be maintained or guided to expand by removing hard acrylic on the buccal side while adding soft acrylic on the palatal side. For BCLP, hard acrylic is removed at the palatal side of the premaxilla and buccal sides of the lateral alveolar segments to allow the premaxilla to be retracted and the lateral alveolar segments to expand.^7^ Soft acrylic is added on the labial side of the premaxilla and palatal aspects of the lateral alveolar segments to reinforce the movements. The nasal stent(s) made of 0.036″ stainless steel wire(s) and acrylic bulb(s) are attached to the plate when the cleft is reduced to about 5 mm. They are modified with soft denture liner to shape the alar cartilage.^8^ Treatment is carried out for at least 10 to 14 weeks in preparation for the infant's primary lip surgery.

Nasoalveolar molding has been shown to reduce the width of the alveolar cleft, elevate the nasal tip, and reshape the nasal cartilage prior to primary lip surgery.^4,6,9^ Although NAM produces successful clinical outcomes, there are several shortcomings including aspiration risk of impression materials,^10^ laborious lab and chairside work, and a large burden of care.^11,12^ The field of PSIO is advancing using computer-aided design and computer-aided manufacturing (CAD/CAM) technology for conducting NAM. The general CAD/CAM NAM concept involves obtaining a digital maxillary model and virtually manipulating it to design a series of models, and/or a series of NAM plates. The plates are then manufactured and worn consecutively by infants to facilitate alveolar molding. Different centers worldwide have undertaken unique CAD/CAM methods to perform NAM. However, a comprehensive overview of these methods has yet to be reported in the literature. This scoping review aims to identify and describe the characteristics of CAD/CAM methods for conducting NAM for infants with CLP based on a structured search of current scientific literature.

## Methods

### Protocol and Eligibility Criteria

This review was conducted following the PRISMA extension for Scoping Reviews (PRISMA-ScR). The protocol was registered on Open Science Framework (https://osf.io/8w579). Articles were selected that fulfilled the following inclusion criteria:

1. Infants with unrepaired, nonsyndromic, complete UCLP or BCLP
2. PSIO using CAD/CAM methods for NAM
3. Descriptive studies, case series, case–control studies, pilot studies, randomized/nonrandomized controlled trials, or cohort studies
4. Written in the English language

The exclusion criteria were:

1. Studies that did not include the study population and intervention
2. Case reports (n = 1), letters to the editor, or systematic reviews
3. Written in languages other than English

### Information Sources and Search

Literature searches were conducted on OVID MEDLINE, OVID Embase, Web of Science, Scopus, and Cochrane Library. Systematic search strategies were developed for each database (see Supplemental 1). Grey literature was searched by entering keywords on Google and CADTH (Canada). All searches were conducted by an academic librarian (MZ) on October 19, 2022, without restrictions for the publication years. The articles were retrieved and exported into EndNote.

### Selection of Sources of Evidence

Two independent reviewers (BKN and CC) conducted Level 1 screening of titles and abstracts to identify relevant articles. Level 2 screening of full text articles was then performed to select eligible articles based on the inclusion and exclusion criteria. Questionnaires were developed and pilot tested on 10 articles for Level 1 and Level 2 screening prior to implementation (see Supplemental 2). The online review software Covidence was used during the screening processes. Any disagreements that arose were resolved in discussion with a third reviewer (KS).

### Data Charting and Data Items

Information about article characteristics, study characteristics, CAD/CAM NAM methods, and outcomes were extracted from the articles by BKN and verified by CC. These characteristics included: author names, year of publication, title, journal, country of origin, objectives, study design, population, and sample size. Information extracted about the CAD/CAM NAM methods were software names, virtual modeling protocols, manufacturing protocols, and materials and equipment.

### Synthesis of Results

The results of all studies included in this scoping review are presented in multiple formats. The outcomes of the screening processes are demonstrated in a PRISMA flow diagram. The characteristics of the studies and CAD/CAM NAM methods are described narratively in the text, depicted in figures and summarized in the table. Where applicable, the methods are presented separately for UCLP and BCLP groups.

## Results

### Selection of Sources of Evidence

**Figure 1.**
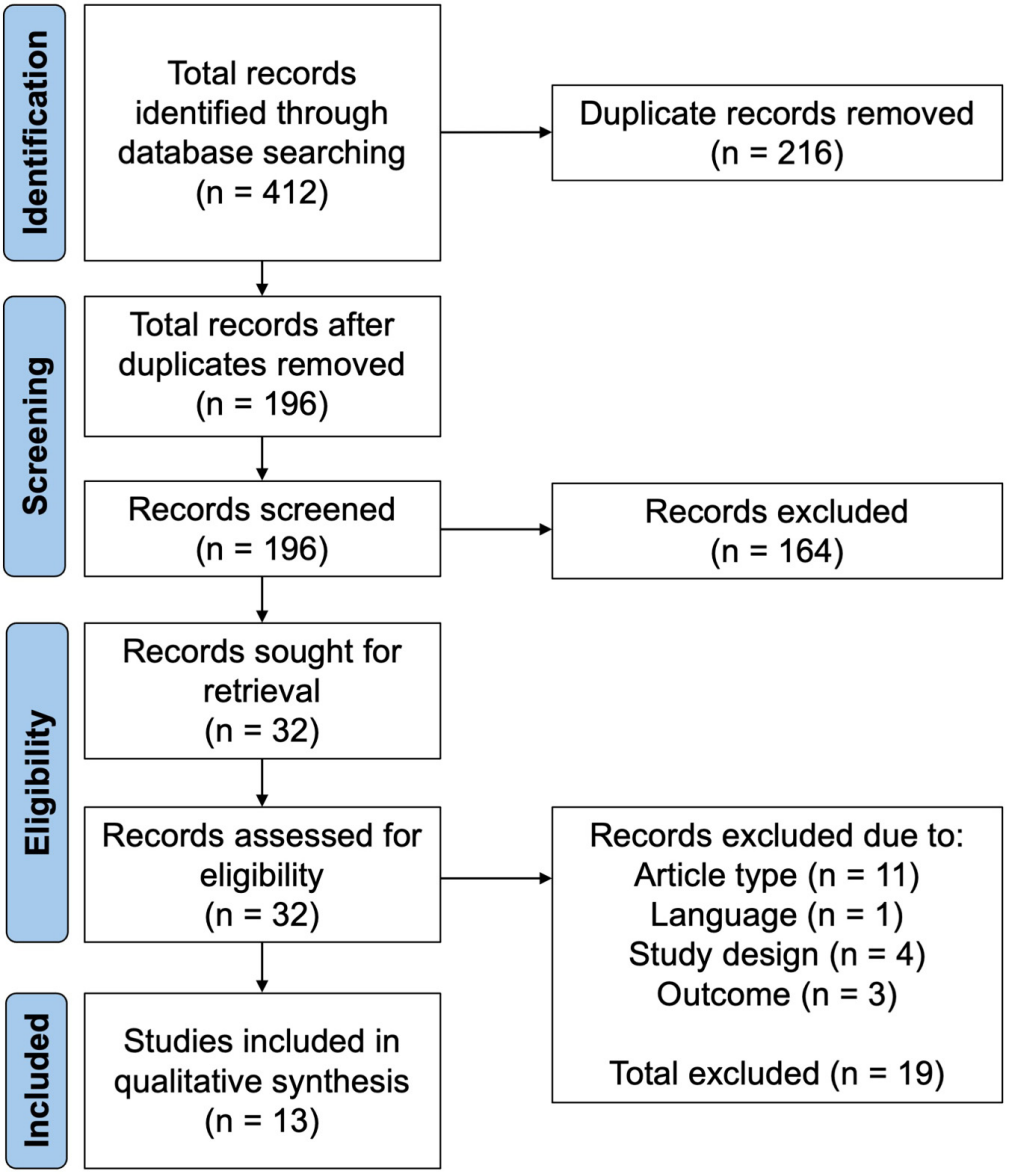
PRISMA flow diagram showing the movement of articles during the screening and selection processes.

A total of 412 articles were retrieved from our searches in the following databases: Medline (69), Embase (92), Web of Science (76), Scopus (148), and Cochrane Library (27). No articles were retrieved in our grey literature searches. A high interrater agreement above 80% between BKN and CC was achieved for the Level 1 and Level 2 screening questionnaires during pilot testing, which demonstrated good consistency among reviewers. After removing 216 duplicate articles, 196 articles underwent Level 1 screening. Disagreements on 15 articles were resolved by KS, resulting in 164 articles being excluded. There were 32 articles retrieved and assessed for eligibility during Level 2 screening. Disagreements on 2 articles were resolved by KS. Ultimately, 19 articles were excluded leading to 13 articles being included in this scoping review. A summary of the screening and selection processes are shown in Figure 1.

### Characteristics of Sources of Evidence

The 13 selected articles were published between 2011 and 2023. Six articles were published by groups from China, 3 from Germany, 3 from Egypt, and one from India. All studies utilized CAD/CAM technology for NAM to treat infants with UCLP and/or BCLP. They encompassed various study designs and sample sizes ranging from 4 to 21 infants per group.

**Figure 2.**
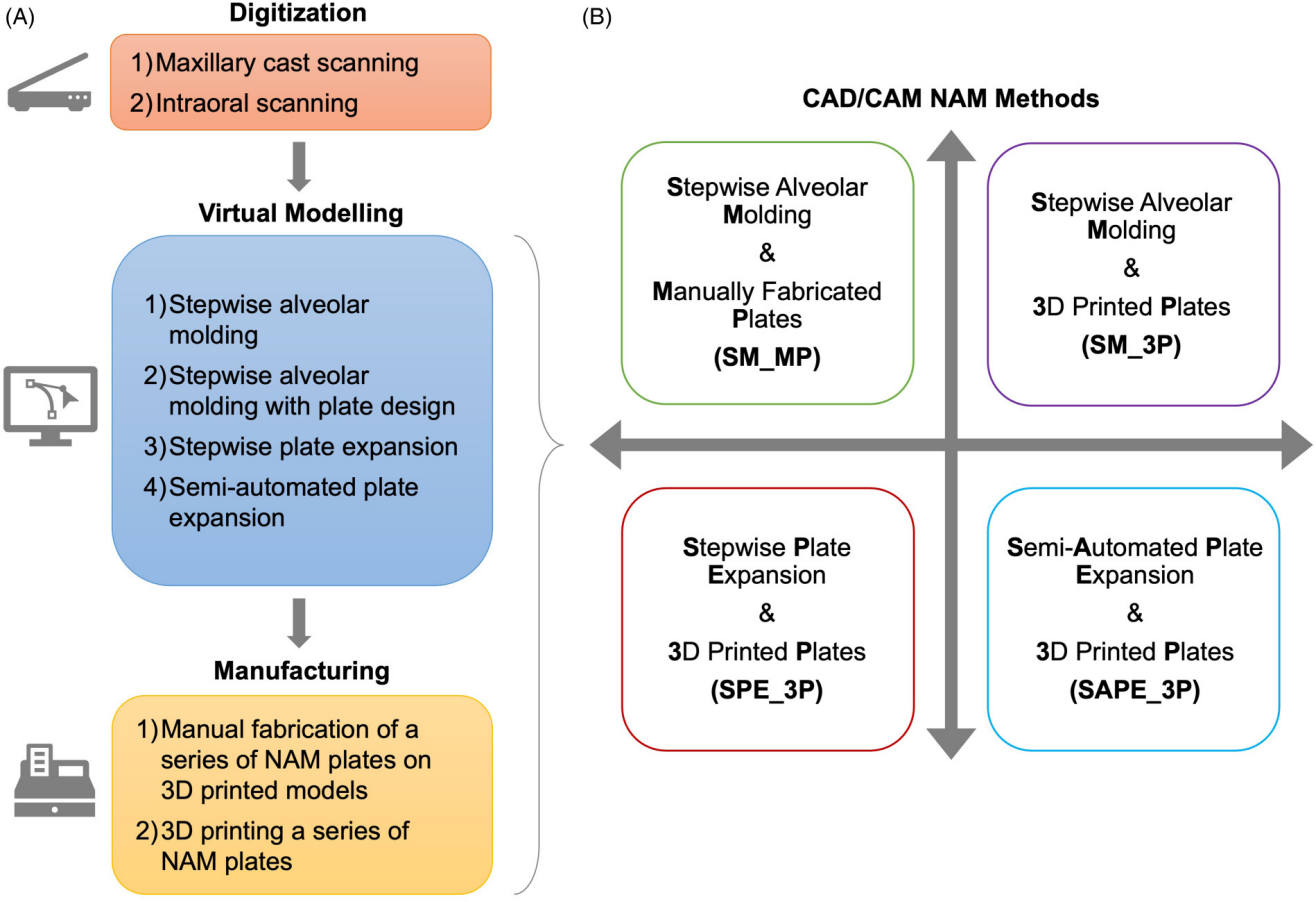
(A) Overview of the computer-aided design and computer-aided manufacturing (CAD/CAM) NAM workflow. (B) Characterization of CAD/CAM nasoalveolar molding (NAM) methods based on the virtual modeling and manufacturing techniques employed.

A variety of CAD/CAM NAM workflows have been reported that incorporated different techniques during the digitization, virtual modeling, and manufacturing stages. In the digitization stage, a digital maxillary model would be acquired through cast model scanning or intraoral scanning. The standard tessellation language (STL) file containing the scan of the maxillary model would be imported into various CAD/CAM software for virtual modeling. Four virtual modeling techniques were identified. The stepwise alveolar molding technique involved designing step-by-step movements of the alveolar segments to approximate the cleft and obtain the desired arch form. This resulted in a series of virtual models incorporating the planned movements. The stepwise alveolar molding with plate design technique was more digitally advanced, whereby a series of virtual plates would be created based on the generated models. The other virtual modeling techniques involved designing a series of consecutively expanded plates derived from the initial virtual model. This process would be performed manually in the stepwise plate expansion technique, or with software algorithms in the semi-automated plate expansion technique. Afterward, the STL file containing the virtual models or plates would be exported for 3D printing. The manufacturing stage involved manually fabricating a series of NAM plates on the 3D-printed models, or directly 3D printing a series of NAM plates. These plates were applied clinically with goals of aligning the alveolar segments, reducing the cleft(s), and creating a harmonious arch form. An overview of the CAD/CAM NAM workflow is shown in Figure 2A.

Four CAD/CAM NAM methods were characterized based on the virtual modeling and manufacturing techniques employed (Figure 2B). These methods were defined as: stepwise alveolar molding and manually fabricated plates (SM_MP); stepwise alveolar molding and 3D-printed plates (SM_3P); stepwise plate expansion and 3D-printed plates (SPE_3P); and semi-automated plate expansion and 3D-printed plates (SAPE_3P). We will describe in detail the characteristics of each CAD/CAM NAM method in the following section.

## Results of Individual Sources of Evidence

### Stepwise Alveolar Molding and Manually Fabricated Plates

The SM_MP method was published in descriptive case series studies and quantitative nonrandomized studies to treat infants with UCLP^13–16^ and BCLP^17,18^ beginning in the early 2010s. The workflow for the SM_MP method involved primarily cast model scanning using a desktop laser scanner, and more recently intraoral scanning using a handheld intraoral scanner. The STL files of the digital model scans were then imported into various CAD/CAM software to simulate alveolar molding.

**Figure 3.**
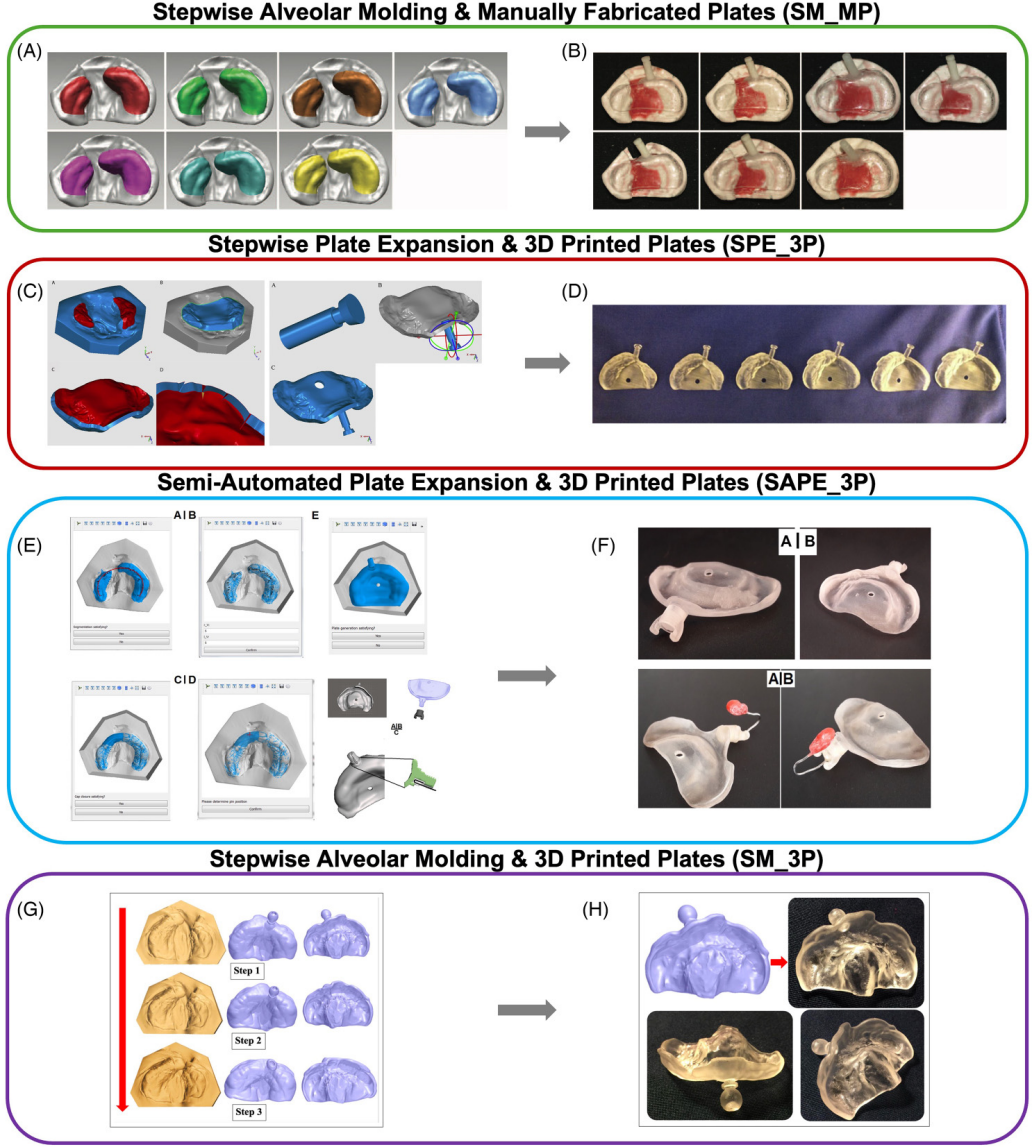
Overview of the workflow for each computer-aided design and computer-aided manufacturing (CAD/CAM) nasoalveolar molding (NAM) method used to treat infants with unilateral cleft lip and palate (UCLP). Stepwise alveolar molding and manually fabricated plates (SM_MP) method. (A) The greater alveolar segment on the maxillary model is moved stepwise in stages to close the cleft resulting in a series of virtual models. (B) A series of NAM plates are manually fabricated from the 3D-printed models. (Adapted with permission from Shen et al.^15^. Published by Wolters Kluwer Health, Inc., 2015.) Stepwise plate expansion and 3D-printed plates (SPE_3P) method. (C) Maxillary seating surfaces are selected, and the cleft is bridged to create an intact maxillary surface. An initial plate is designed by thickening the surface, incorporating a retention arm and a ventilation hole. A series of six plates would be designed stepwise by applying a 3% growth factor consecutively. (D) A series of six 3D-printed plates. (Adapted with permission from Ritschl et al.^19^. Published by Elsevier, 2016.) Semi-automated plate expansion and 3D-printed plates (SAPE_3P) method. (E) An algorithm automatically detects the alveolar ridge on the initial maxillary model. The cleft is bridged, and the position of the retention arm is determined. A virtual plate is designed as a thickened shell over the maxillary surface, and a retention arm and ventilation hole are incorporated. A retention nut is also designed to fit over the retention arm to secure the nasal stent and allow for buccal tapings. (F) Views of a 3D-printed plate without and with a nasal stent attached. (Adapted from Grill et al.^20,^^21^, licensed under CC BY 4.0: http://creativecommons.org/licenses/by/4.0/.) Stepwise alveolar molding and 3D-printed plates (SM_3P) method. (G) A series of NAM plates with retention arms are virtually constructed over models that incorporated stepwise alveolar molding movements. (H) 3D-printed NAM plate based on the virtually designed plate. (Adapted with permission from Abd El-Ghafour et al.^25^. Published by Sage Publications, 2020).

Virtual modeling for UCLP involved dividing the digital maxillary model into several parts to move the greater segment in 8 to 10 steps,^13–15^ or 15 steps,^16^ to align and approximate the alveolar segments. The greater segment was either rotated and/or translated to reduce the cleft by 1 mm each week.^15,16^ This generated a series of virtual models that incorporated stages of stepwise alveolar molding movements (Figure 3A). The virtual models were exported from CAD/CAM software to be 3D printed. A series of NAM plates were manually fabricated on the 3D-printed models using a combination of hard and soft acrylic resins^13–15^ or thermoplastic material (Figure 3B).^16^ These prefabricated plates were provided to the infant's parents to change weekly. Nasal molding for UCLP was done with a stent made from 0.036″ stainless steel wire and a soft resin or silicone bulb according to conventional NAM protocol. It was performed when the cleft was narrowed to between 3 mm and 5 mm,^13,15^ or after 3 to 4 weeks of treatment initiation.^13–15^ The plates were secured using orthodontic elastics (0.25″, 3.5 oz) and adhesive tapes. All follow-up visits were conducted monthly.

**Figure 4.**
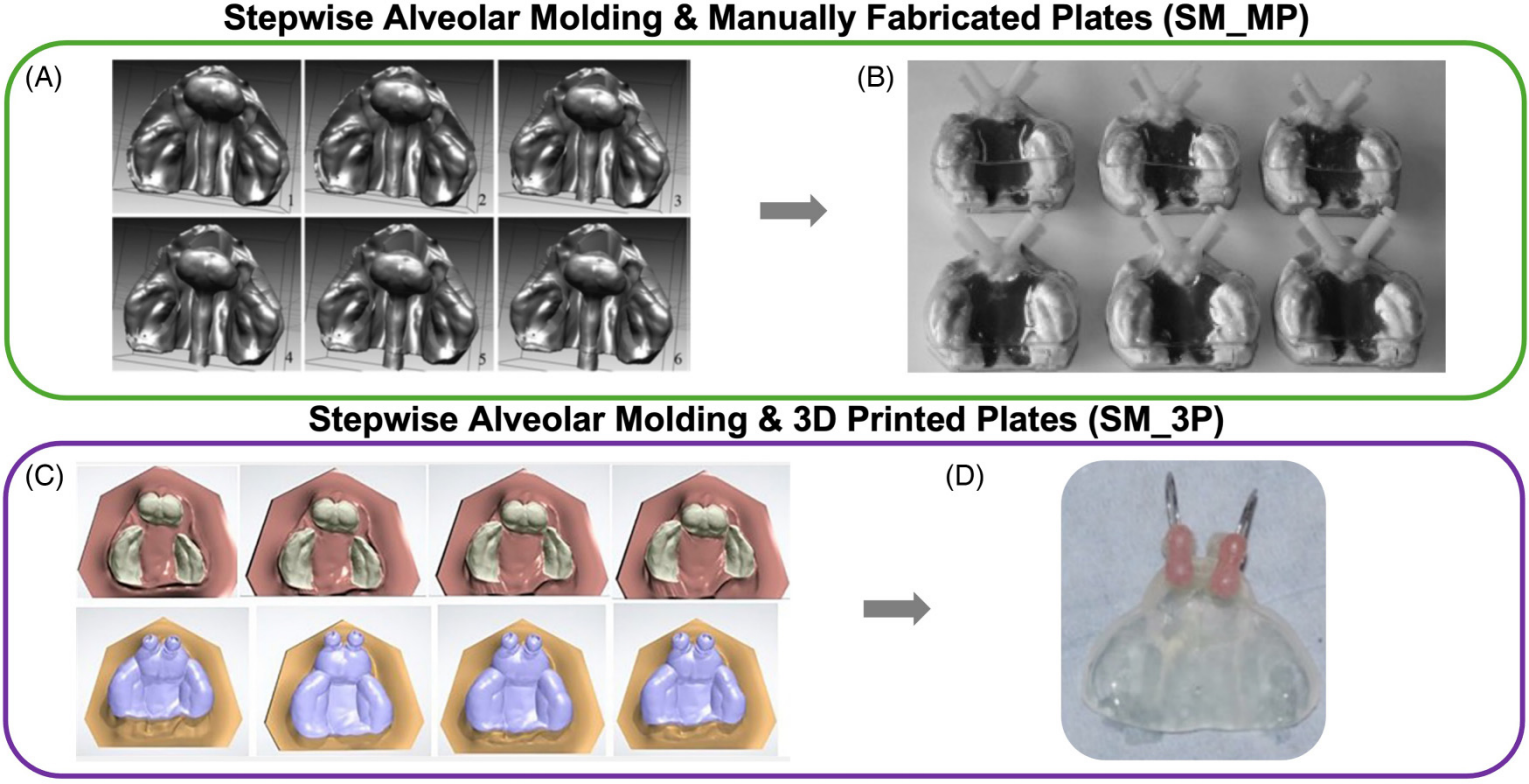
Overview of the workflow for each computer-aided design and computer-aided manufacturing (CAD/CAM) nasoalveolar molding (NAM) method used to treat infants with bilateral cleft lip and palate (BCLP). Stepwise alveolar molding and manually fabricated plates (SM_MP) method. (A) The premaxilla is retracted, and the lateral alveolar segments are moved outward stepwise in stages, generating a series of virtual models. (B) A series of NAM plates are fabricated manually over the 3D-printed series of models. (Adapted with permission from Gong et al.^17^. Published by Elsevier, 2012.) Stepwise alveolar molding and 3D-printed plates (SM_3P) method. (C) A series of virtual models are created from stepwise retraction of the premaxilla and rotation and displacement movements of the lateral alveolar segments. A series of NAM plates are virtually constructed over the virtual models. (Adapted with permission from El-Ashmawi et al.^26^. Published by Sage Publications, 2022.) (D) A 3D-printed plate with two nasal stents attached. (Adapted with permission from El-Ashmawi et al.^27^. Published by Sage Publications, 2023).

For cases of BCLP, the digital maxillary model was divided into 3 segments (premaxilla and 2 lateral alveolar segments) and moved in 12 to 20 steps.^17,18^ The posterior lateral alveolar segments were moved outward, whereas the premaxilla was retracted and derotated (Figure 4A).^17^ The amount of movement depended on clinical judgement and the severity of the initial cleft. A series of virtual models were created, and 3D printed. A series of NAM plates (2 mm thick) were fabricated manually on the models, and 2 retentive buttons were added on the plates (Figure 4B). The clinical protocol for BCLP was like UCLP except that nasal molding was performed when the premaxilla was retracted to the ideal position.^17,18^

### Stepwise Plate Expansion and 3D-Printed Plates Method

The SPE_3P method was reported in a nonrandomized pilot study conducted in 2016 involving infants with UCLP divided into an intervention group and comparison NAM group.^19^ The workflow involved scanning stone maxillary cast models using a benchtop scanner and exporting the STL files to CAD/CAM software. The virtual modeling stage involved designing a series of NAM plates by stepwise expansion of an initial plate created from the original digitized maxillary model.^19,20^ First, the seating surfaces of the greater and lesser alveolar segments were selected on the model to allow the cleft to be virtually bridged. The intact maxillary surface was thickened to create a 2 mm shell for the virtual plate and the edges were smoothened. A retention arm was positioned at the middle of the mesial third of the cleft lip 40° downward to the occlusal plane. Finally, a 4 mm vent hole was created 5 mm anterior to posterior border of the plate (Figure 3C). The resultant plate was expanded consecutively by a 3% empirical growth factor to allow room for expansion of the alveolar ridge with successive plates. In total, 6 plates were designed and then 3D printed (Figure 3D). The set of consecutively expanded NAM plates were employed clinically. A nasal stent composed of a stainless-steel wire and resin bulb was added chairside to the plate for nasal molding when the cleft was narrowed to less than 6 mm.

### Semi-Automated Plate Expansion and 3D-Printed Plates Method

The SAPE_3P method, introduced as RapidNAM in 2018, was investigated in a nonrandomized pilot study where infants with UCLP were either treated with this method or the SPE_3P method.^20,21^ Although the digitization protocols were similar among the 2 methods, the virtual modeling approach in the SAPE_3P method was more advanced. This method employed a graphical user interface (GUI) to design a series of consecutively enlarged NAM plates with a novel click-in nasal stent system.^20,21^ The alveolar segments would be demarcated on the digital model as curves along the buccal and palatal sides. An ellipse-finding algorithm automatically detected the alveolar ridge. The cleft was digitally filled by extruding the alveolus from the cross section of the greater alveolar segment toward the lesser alveolar segment.^22^ Following closure of the cleft, the position of the retention arm was determined. A virtual plate was then designed over the maxillary surface. It included a ventilation hole, as well as a retention arm containing a retention groove and threads (Figure 3E). The nasal stent was designed to be inserted in the retention groove and secured to the arm using a retention nut. The resultant plates were expanded by a monthly percentage relative growth factor reflecting postnatal dentoalveolar and palatal growth in healthy newborns.^23^ These plates were then 3D printed and delivered chairside (Figure 3F). Adhesive cream was used for plate retention. Buccal taping and lip taping were also performed. Follow-up visits were done weekly where plates were changed or adjusted as necessary by adding or removing acrylic at the cleft lip vermillion border for comfort. It was found that 3 to 4 plates were required to be used out of the 6 prefabricated plates.

### Stepwise Alveolar Molding and 3D-Printed Plates Method

The SM_3P method was investigated in a case series study^24^ and 2 quantitative randomized controlled trials in the early 2020s.^25–27^ The case series study included both infants with UCLP and BCLP.^24^ One randomized controlled trial involved infants with UCLP treated with the SM_3P method versus untreated infants,^25^ whereas the other focused on infants with BCLP treated with the SM_3P method versus conventional NAM.^26,27^ In this method, digitization of the maxillary model was done through cast model scanning with a desktop scanner, or intraoral scanning with a handheld intraoral scanner. Virtual modeling was performed on CAD/CAM software in 2 stages: (1) modification of the digital model via stepwise alveolar movements, and (2) design of a series of NAM plates based on virtual models that incorporated the alveolar movements.

For UCLP, the digital maxillary model was divided into 3 segments (2 posterior lateral segments and a middle premaxillary segment). The segments were moved through translation and rotation steps <1 mm weekly,^24^ or according to personal judgment,^25^ until the desired arch form and reduction of the cleft were achieved. Movements were grouped to be done at weekly^24^ or monthly intervals,^25^ which created a series of virtual models. The virtual models could also be widened by about 1 mm per month to accommodate maxillary growth. A series of NAM plates with a retention arm were then virtually constructed on CAD/CAM software as 2 mm thick shells on the series of virtual models (Figure 3G). Finally, the plates were 3D printed using a variety of 3D printers and 3D printing resins (Figure 3H). Denture adhesive cream aided with retention of the plate, and lip taping was performed.^25^ In one study, patients came for biweekly follow-up visits and plates were changed every month by clinicians.^25^ In contrast, in another study parents were responsible for changing plates every week and came for monthly follow-up visits.^24^

Virtual modeling for BCLP also involved using CAD/CAM software to segment the maxillary model into 3 parts (2 lateral alveolar segments and premaxilla) and move them accordingly into alignment. The centers of rotation of each part were first identified. The lateral alveolar segments were designed to rotate 5° outward, displace 2 mm anteriorly, and 1 mm outward. The premaxillary segment was designed to rotate 1° and tip 1°.^26^ The planned movements were grouped into stages giving rise to a series of virtual models. These models were then used as templates to design a series of virtual plates with an offset of 1 mm and 2 retention arms (Figure 4C). The virtual plates were then 3D printed (Figure 4D). In one study, patients came for weekly follow up visits and the NAM plates were changed biweekly.^26,27^ In the other study, parents were instructed to change the plates at home weekly.^24^ A prolabial tape was used to lengthen the columella, and elastics and tapes were used for retention. Table 1 summarizes the characteristics of the CAD/CAM NAM methods performed in all studies included in this review.

**Table 1.** Characteristics of computer-aided design and computer-aided manufacturing (CAD/CAM) nasoalveolar molding (NAM) methods reported in all included studies for infants with unilateral cleft lip and palate (UCLP) and bilateral cleft lip and palate (BCLP). ^a^

CAD/CAM NAM Method	Population	Author Year Origin	Scanner	Digitization Technique	CAD/CAM Software	CAD/CAM NAM Virtual Modeling Protocol	Manufacturing Materials & Equipment
**SM_MP**	UCLP	Yu et al^13^China	Vivid 910 (Konica Minolta Holdings, Inc, Tokyo, Japan)	Cast scanning	Rapid Form 2006 (Inus Technology, Inc, Seoul, South Korea)	Digital maxillary model was split into several regions to move the greater segment in 8-10 steps to align and approximate the segments, reduce the cleft, correct the midline, and maintain lateral width of segments. Digital data were exported to 3D print a series of scaled models.	A series of 2 mm thick plates with a retention arm were fabricated using the 3D-printed models Materials and equipment were not specified
		Yu et al^14^ China	Vivid 910 (Konica Minolta Holdings, Inc, Tokyo, Japan)	Cast scanning	Rapid Form 2006 (Inus Technology, Inc, Seoul, South Korea)	Digital maxillary model was divided and modified in several steps. Digital data were exported to 3D print a series of scaled models.	A series of plates with a retention arm were fabricated based on the 3D-printed models Materials and equipment were not specified
		Shen et al^15^China	Vivid 910 (Konica Minolta Holdings, Inc, Tokyo, Japan)	Cast scanning	Rapid Form 2006 (Inus Technology, Inc, Seoul, South Korea)	Greater alveolar segment was rotated to reduce the cleft by 1 mm each week in 8 to 10 steps. Maxillary models of the predicted molding stages were 3D printed.	3D printer to create models A series of plates with a retention arm were fabricated based on the 3D-printed models Hard and soft acrylic resins were used for the plates
		Batra et al^16^ India	3Shape TRIOS (3Shape, Copenhagen, Denmark)	Intraoral scanning	3Shape Ortho Analyzer 3Shape OrthoPlanner (3Shape A/S, Copenhagen, Denmark)	The greater and lesser alveolar segments were separated, and an ovoid arch shape was created using 3 points on the maxillary model as a guide for the ideal arch form. The greater segment was moved by 1 mm at most per stage to reduce the cleft, correct the midlines and maintain lateral width of the segments. A total of 15 movement stages were set up and exported to print a series of models.	3D Printer: Eden 500 (Stratasys, Rehovot, Israel) 3D printing resin: MED620 (Stratasys, Rehovot, Israel) Triple-layer, resilient-plastic material: OrthoAligner Ultimate (Compass 3D, Belo Horizonte, Brazil) 0.66 mm thickness
	BCLP	Gong et al^17^ China	Vivid 910 (Konica Minolta Holdings, Inc, Tokyo, Japan)	Cast scanning	Rapid Form 2006 (Inus Technology, Inc, Seoul, South Korea)	Digital maxillary model was divided into 3 segments to move the posterior lateral alveolar segments outward, and retract and derotate the premaxilla, in 12-16 steps. Digital data were exported to 3D print a series of scaled models.	A series of 2 mm thick plates with 2 retention arms were fabricated using the 3D-printed models Materials and equipment were not specified
		Gong et al^18^China	Vivid 910 (Konica Minolta Holdings, Inc, Tokyo, Japan)	Cast scanning	Rapid Form XOR3 (Inus Technology, Inc, Seoul, South Korea)	CAD/CAM NAM procedure was designed in 16-20 steps. A series of scaled models were 3D printed.	A series of 2 mm thick plates with 2 retention arms were fabricated using the 3D-printed models Materials and equipment were not specified
**SPE_3P**	UCLP	Ritschl et al^19^ Germany	3Shape D700, (3Shape, Copenhagen, Denmark)	Cast scanning	Geomagic^©^ Studio 12 and Qualify 12 (Geomagic)	Maxillary seating surface regions were defined and “Bridge” function was used to fill the cleft. Refinements were performed and the whole surface was thickened to 2 mm using the “Shell Function” forming the plate. Retentive pin was designed and positioned in the middle of the mesial third of the cleft lip and oriented 40° downward to the occlusal plane. 4 mm vent hole was created 5 mm anterior to posterior border of palate. Six plates were designed and have integrated growth factor of 3% in sagittal and transverse planes (total 18%) for plate expansions.	Data exported to 3D print using DLP technique (slice thickness 0.1 mm) by 3D Labs GmbH (St. Georgen, Germany) Material: methacrylate-based biocompatible material (Innovation MediTech GmbH, Unna, Germany)
**SAPE_3P**	UCLP	Grill et al^20^ Germany	3Shape D500, (3Shape)	Cast scanning	Geomagic^©^ Studio 12 and Qualify 12 (Geomagic) RapidNAM-GUI software system	CAD protocol for SPE_3P (CAD/CAM NAM) group as described previously by Ritschl et al For SAPE_3P (RapidNAM) group, a series of 6 plates was designed through a graphical user interface (GUI) that involved: automated detection of the alveolar crest using ellipse-finding algorithm, manual selection of bridging areas on the greater and lesser segments, virtual gap closure, determining pin position, smoothing of the plate surface. A monthly percentage relative growth rate was applied to the plates.	Data exported to 3D print using DLP technique (slice thickness 0.1 mm) by 3D Labs GmbH Material: methacrylate-based biocompatible material (Innovation MediTech GmbH)
		Grill et al^21^ Germany				CAD protocol for SPE_3P (CAD/CAM NAM) group as described previously by Ritschl et al. (2016) As described previously for SAPE_3P (RapidNAM) group (Grill et al. 2018) for plate design. Retention pin was designed on the plate at 40° tilt. It had a screw thread design and retention groove for the nasal stent wire. A corresponding nut with 2 retentions for buccal taping was designed to secure the stent.	
**SM_3P**	UCLP BCLP	Gong et al^24^ China	3Shape TRIOS (Copenhagen, Denmark)	Intraoral scanning	Geomagic Design X 2016 (3D Systems, Inc.) Rhino (Rhinoceros 3D Version 5.0 Robert McNeel & Associates)	Stepwise Alveolar Molding: Digital maxillary model was modified by subdividing it into segments (2 posterior lateral alveolar segments and the middle premaxillary segment). Segments were moved to narrow the cleft <1 mm per week via translation and rotation movements. Models were widened 1 mm per month to accommodate growth. Plate Design: A series of NAM plates were constructed from the series of virtual models for each movement stage.	Data exported to 3D print plates (2 mm in thickness) 3D Printer: Objet30 OrthoDesk (Stratasys) Resin material: Biocompatible MED610 (Stratasys)
	UCLP	Abd El-Ghafour et al^25^Egypt	3Shape R500 (3Shape)	Cast scanning	3Shape Scan-it Manager 3Shape Ortho Analyzer 3Shape Appliance Designer (3Shape A/S)	Stepwise Alveolar Molding: Digital maxillary model was divided into 3 segments (2 posterior lateral alveolar segments and the middle premaxillary segment). Three segments were moved using control panel until desired arch form was designed. Movements were separated into 3 stages. Plate Design: A series of NAM plates were constructed based on the series of virtual models. The models had to be digitally relieved in the cleft area and undercuts and reduced at pressure areas. The path of insertion was selected with retention specified at 0.1 mm. The plates were constructed using shell construction feature and thickened to 2 mm. Button was virtually added and positioned.	Data exported to 3D print plates (2 mm in thickness) using stereolithography technique ZENITH U 3D printer, Desktop system (Dentis) Acrylic resin material: NextDent Ortho Rigid (Vertex-Dental)
	BCLP	El-Ashmawi et al^26^ Egypt	3Shape R500 (3Shape)	Cast scanning	3Shape Ortho System (3Shape A/S)	Stepwise Alveolar Molding: Three landmarks were identified on the maxillary model (incisal papilla, right, and left tuberosities points) to identify the center of rotations of the premaxilla and lateral alveolar segments. Lateral alveolar segments were designed to rotate 5° outward, displace 2 mm anteriorly and 1 mm outward within each stage. The premaxilla was designed to rotate 1° and tip 1° at most within each stage. To account for growth, the edges of the alveolar margins of the lateral segments and occlusal side of the premaxilla were digitally rounded. Plate Design: A plate was constructed for each movement stage with a 1 mm offset and 2 retention buttons.	Data exported to 3D print plates (2 mm in thickness) ZENITH stereolithography printer (Dentis) Resin material: ZMD-1000B Clear-SG (Dentis)
		El-Ashmawi et al^27^ Egypt					

a Abbreviations: BCLP, bilateral cleft lip and palate; CAD/CAM, computer-aided design and computer-aided manufacturing; NAM, nasoalveolar molding; SM_MP, stepwise alveolar molding and manually fabricated plates; SPE_3P, stepwise plate expansion and 3D-printed plates; SAPE_3P, semi-automated plate expansion and 3D-printed plates, SM_3P, stepwise alveolar molding and 3D-printed plates; UCLP, unilateral cleft lip and palate.

## Synthesis of Results

### Overview of CAD/CAM NAM Methods

**Figure 5.**
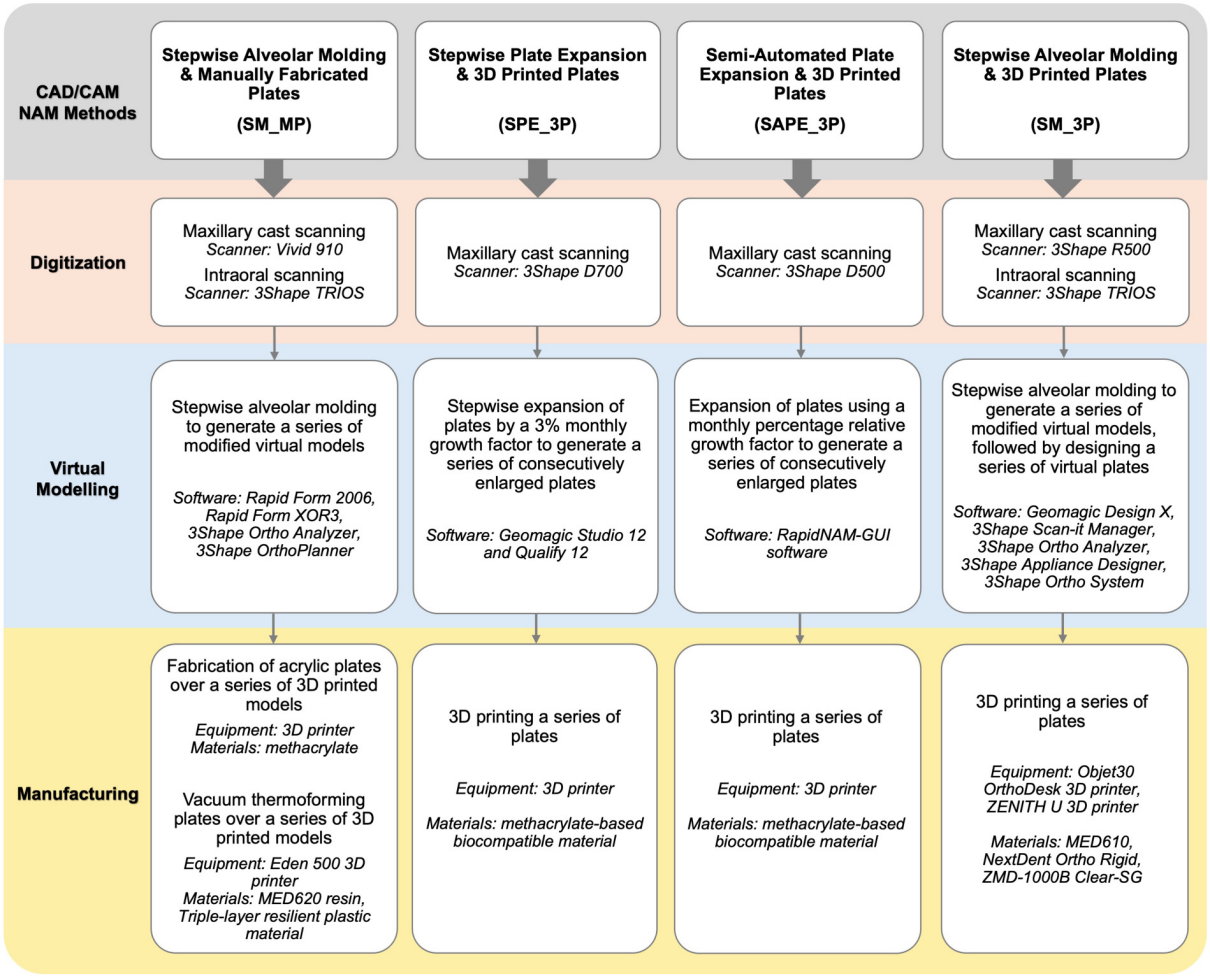
Summary of computer-aided design and computer-aided manufacturing (CAD/CAM) nasoalveolar molding (NAM) methods and their associated workflows.

The 4 CAD/CAM NAM methods we characterized, along with their unique workflows, are summarized in Figure 5. All CAD/CAM NAM methods employ cast model scanning for digitization of the maxilla. Intraoral scanning can also be used, although it has only been explored in the SM_MP and SM_3P methods. For virtual modeling, the SM_MP and SM_3P methods incorporate stepwise alveolar molding, whereas the SPE_3P and SAPE_3P methods incorporate stepwise plate expansion. Stepwise alveolar molding simulates movements of the alveolar segments to harmonize the arch form and reduce the size of the cleft(s) in stages, thereby generating a series of virtual models. The SM_3P method requires further designing a series of virtual NAM plates based on the models. Stepwise plate expansion involves digitally bridging the cleft on the maxillary model and designing a virtual NAM plate as a thickened shell over the intact surface. In the SPE_3P method, this process is carried out manually and the initial plate is expanded step-by-step by a factor of 3% to generate a series of consecutively enlarged plates. The SAPE_3P method follows a similar process using a GUI with a built-in algorithm to expedite the virtual design process, and a monthly percentage relative growth factor to create a series of consecutively enlarged plates. Manufacturing a series of NAM plates following the SM_MP method occurs manually using acrylic or thermoplastic material over 3D-printed models. In contrast, all other CAD/CAM NAM methods directly 3D print the virtual series of plates using resins.

## Discussion

### Summary of Evidence

Following the search criteria, we evaluated 13 articles based on CAD/CAM NAM, which led to the characterization of 4 methods. The SM_MP method was the most common, followed by the SM_3P, SPE_3P, and SAPE_3P methods. All 4 methods can be used to treat infants with UCLP; however, to our knowledge only the SM_MP and SM_3P methods have been applied to treat infants with BCLP. The SM_MP method has a simpler virtual modeling process compared to the SM_3P method that requires additional steps to virtually design plates following stepwise alveolar molding movements. Furthermore, the familiar plate manufacturing technique as conventional NAM may allow it to be more readily adopted. Disadvantages are that models need to be 3D printed, and lab technicians are required to fabricate plates over these models with acrylic or thermoplastic aligner material. Acrylic plates would be more rigid, durable and easy to adjust chairside, whereas vacuum thermoformed plates would be more flexible and challenging to modify. The SM_3P method is more advanced than the SM_MP method owing to its streamlined process for designing plates and directly 3D printing them. Although it may require more technical expertise and software, it has advantages of reducing waste of 3D-printed models and manual lab work. The SM_MP and SM_3P methods stage stepwise movements of alveolar segment(s) per plate. This may allow for more controlled alveolar movements compared to the SPE_3P and SAPE_3P methods where plates are intended to guide passive growth of the alveolar segments. The SPE_3P method has a simpler virtual modeling protocol that would allow it to be implemented more widely than the SAPE_3P method. The SAPE_3P method requires advanced knowledge of computer programming since a specific GUI was created to execute some automated functions. Consequently, this method is limited to groups that possess the specific GUI, or those with the ability to create their own. The main appeal of the SAPE_3P method is the time saving advantages. It can expedite the virtual fabrication of plates from 1.5 h to 10 to 15 min compared to the SPE_3P method, and it enables efficient chairside exchange of nasal stents between plates.^20,21^ The production costs of 3D-printed plates in the SPE_3P and SAPE_3P methods are reported to be similar and lower than conventional NAM plates.^20^ It can be inferred that the SM_3P method would also have cost saving advantages over the SM_MP method. The shared drawbacks of all methods are that they require CAD/CAM software, technical experience, and accessibility to 3D printers in house or by outsourcing. These may pose barriers limiting the use of CAD/CAM NAM in low-resource countries. Overall, each CAD/CAM NAM method carries unique advantages and disadvantages and may evolve over the years with advancements in technology and material science.

There is a general motivation to implement fully digital CAD/CAM NAM workflows. This will require the use of chairside intraoral scanning rather than cast model scanning that has been predominantly used. Intraoral scanning can be considered a safe alternative to conventional intraoral impressions that carry risks of aspiration and airway obstruction.^10^ A recent study found that intraoral scanning in neonates, infants, and children under 3 years of age with CLP was a safe and feasible procedure with a median scan duration of 2.52 min (range: 0.60-8.95 min).^28^ Furthermore, intraoral scanning offered the convenience of rescanning if there were defects. However, there are still several challenges such as loss of details when scanning across a cleft,^28,29^ keeping infants still during the scanning process, and fitting large scanner heads inside their mouths to capture all maxillary structures.^24,25,28,30^ To overcome issues with scanning across a cleft, it has been proposed to capture parts of the lip or nose, or place a cotton swab in the area, to temporarily bridge the cleft.^28^ This can allow a continuous scan to be captured. Miniaturization of intraoral scanners with fast scanning times may also promote easier adoption of intraoral scanning in infants with CLP.

Full automation of the virtual modeling process in the CAD/CAM NAM workflow is the future direction of the field. To date, the SAPE_3P method is the only CAD/CAM NAM method developed with some automated processes. With its semi-automated protocol, the SAPE_3P method has demonstrated enhanced efficiency in designing a series of NAM plates for UCLP. A limitation is that the algorithm can experience challenges in detecting the alveolar ridge in UCLP cases with broad clefts and possibly less uniform ridges found in BCLP cases.^20^ Therefore, it is important that the operator be involved in some steps of the virtual modeling process to make modifications. Full automation might be able to reduce burden on operators and consequently promote more widespread adoption of CAD/CAM NAM. Future research should focus on developing user-friendly software with well-developed algorithms for virtual modeling of CAD/CAM NAM for UCLP and BCLP cases.

Selection of biocompatible materials to manufacture plates for CAD/CAM NAM is critical. In the early to mid-2010s, NAM plates were made with self-curing acrylic polymethyl methacrylate (PMMA) (eg, Orthocryl (Dentaurum). It has acceptable biocompatibility with oral tissues and is FDA and Health Canada approved.^31,32^ A concern about self-curing PMMA is that unpolymerized MMA can cause inflammation or irritation of oral tissues. However, a study evaluating safety in vitro demonstrated that MMA elution from conventional PMMA aged up to 60 days in solution was lower than the safety limit set by the ISO 20795-1 standards for dental polymers.^33^ In 2020, thermoplastic clear aligner material was used to vacuum thermoform NAM plates on 3D-printed models. Polyethylene terephthalate found in this material is approved by the FDA^32^ and Health Canada and has good biocompatibility.^34^ From the mid-2010s and onward, NAM plates have been 3D printed with resins classified by the FDA as Class I low risk (eg, Fotodent IBT (Innovation MediTech GmbH); MED610 (Stratasys) and NextDent Ortho Rigid (Vertex-Dental)), or Class II intermediate risk (eg, ZMD-1000B Clear-SG (Zenith, Dentis)). Health Canada classifies NextDent Ortho Rigid (Vertex-Dental) as a class II medical device posing low-to-medium risk. Concerns have arisen about the safety of prolonged intraoral usage of MED610 based NAM plates since this material is recommended by Stratasys for temporary mucosal membrane contact up to 24 h.^35^ With a variety of materials available on the market and new ones emerging, it is important that approved biocompatible materials are used for CAD/CAM NAM, and that operators adhere to manufacturers’ recommendations for their intraoral usage.

## Limitations

There are limitations with the findings presented in this scoping review. Research on CAD/CAM NAM is scarce; hence, our inclusion criteria allowed for many types of study designs, as well as both UCLP and BCLP populations. The findings are derived from case series, pilot studies and/or nonrandomized controlled trials, which are low on the hierarchy of evidence. Only 3 randomized controlled trials were included, which provide higher quality of evidence. This highlights a need for more high-quality randomized controlled trials to be conducted on CAD/CAM NAM. There are also limitations with the generalizability of the findings presented in this review. Multicenter collaboration on the same CAD/CAM NAM methods could help standardize the design and manufacturing protocols. We thematically characterized 4 CAD/CAM NAM methods based on the virtual modeling and manufacturing techniques employed. A breakdown of CAD/CAM NAM methods in this manner has not been described previously in the literature. Therefore, other people may have interpretations of CAD/CAM NAM methods that differ from how it has been presented here.

## Conclusions

This scoping review identifies and synthesizes the characteristics of 4 CAD/CAM methods for NAM. Both the SM_MP and SM_3P methods are used to virtually simulate stepwise alveolar movements; however, the SM_3P method exhibits more advanced design and manufacture of plates. The SPE_3P and SAPE_3P methods both employ virtual stepwise plate expansion, although the SAPE_3P method is more advanced owing to its semi-automated protocol. This review could serve as a guide to help centers implement or improve their use of CAD/CAM technology for performing NAM.

## Supplemental Material

sj-docx-1-cpc-10.1177_10556656251363400 - Supplemental material for Computer-Aided Design and Computer-Aided Manufacturing Technology for Conducting Nasoalveolar Molding for Infants With Cleft Lip and Palate: A Scoping Review Supplemental material, sj-docx-1-cpc-10.1177_10556656251363400 for Computer-Aided Design and Computer-Aided Manufacturing Technology for Conducting Nasoalveolar Molding for Infants With Cleft Lip and Palate: A Scoping Review by Bach Kim Nguyen, Camila Caro, Kyle Stevens, Gabriella A Garisto and Yoav Finer in The Cleft Palate Craniofacial Journal
