# Supplementary material for: Computer-Aided Design and Computer-Aided Manufacturing Technology for Conducting Nasoalveolar Molding for Infants With Cleft Lip and Palate: A Scoping Review
Source: Cleft Palate Craniofac J. 2025 Sep 11;63(8):2698–712. doi: 10.1177/10556656251363400 (PMC13334062; doi:10.1177/10556656251363400)
Supplement: sj-docx-1-cpc-10.1177_10556656251363400 - Supplemental material for Computer-Aided Design and Computer-Aided Manufacturing Technology for Conducting Nasoalveolar Molding for Infants With Cleft Lip and Palate: A Scoping Review [file sj-docx-1-cpc-10.1177_10556656251363400.docx]

***1******. Search Strategies***

**Ovid MEDLINE**

| **#** | **Searches** | **Results** |
| --- | --- | --- |
| 1 | Cleft Lip/ | 16615 |
| 2 | Cleft Palate/ | 22361 |
| 3 | (cleft adj2 (lip* or palate)).tw,kf. | 24525 |
| 4 | exp Computer-Aided Design/ | 27390 |
| 5 | ("computer aided" adj2 (design or manufacturing)).tw,kf. | 6753 |
| 6 | ("computer assisted" adj2 (design or manufacturing)).tw,kf. | 943 |
| 7 | (CAD-CAM or "CAD/CAM").tw,kf. | 5294 |
| 8 | Printing, Three-Dimensional/ | 10589 |
| 9 | ((three dimension* or "3 dimension*" or 3D or 3-D) adj2 print*).tw,kf. | 22194 |
| 10 | (reverse adj2 engin*).tw,kf. | 2045 |
| 11 | model*.tw,kf. | 3545513 |
| 12 | ((digital or virtual) adj2 (workflow or design or techniqu* or method*)).tw,kf. | 9892 |
| 13 | ("nasoalveol* molding" or NAM or "palatal obturator*" or "nasoalveolar therap*" or dNAM or pNAM).tw,kf. | 5728 |
| 14 | ("presurgical infant orthop?edics" or PSIO or "preoperative orthop?edics" or "pre-operative orthop?edics").tw,kf. | 132 |
| 15 | Orthopedics/ | 23338 |
| 16 | Preoperative Care/ | 65042 |
| 17 | 1 or 2 or 3 | 31842 |
| 18 | 4 or 5 or 6 or 7 or 8 or 9 or 10 or 11 or 12 | 3582855 |
| 19 | 15 and 16 | 213 |
| 20 | 13 or 14 or 19 | 6038 |
| 21 | 17 and 18 and 20 | 69 |

**Embase**

| **#** | **Searches** | **Results** |
| --- | --- | --- |
| 1 | Cleft Lip/ | 18316 |
| 2 | Cleft Palate/ | 28626 |
| 3 | (cleft adj2 (lip* or palate)).tw,kf. | 31019 |
| 4 | exp computer aided design/ | 45400 |
| 5 | ("computer aided" adj2 (design or manufacturing)).tw,kf. | 7293 |
| 6 | ("computer assisted" adj2 (design or manufacturing)).tw,kf. | 1058 |
| 7 | (CAD-CAM or "CAD/CAM").tw,kf. | 5151 |
| 8 | three dimensional printing/ | 19835 |
| 9 | ((three dimension* or "3 dimension*" or 3D or 3-D) adj2 print*).tw,kf. | 24485 |
| 10 | (reverse adj2 engin*).tw,kf. | 2368 |
| 11 | model*.tw,kf. | 4495747 |
| 12 | ((digital or virtual) adj2 (workflow or design or techniqu* or method*)).tw,kf. | 12497 |
| 13 | ("nasoalveol* molding" or NAM or "palatal obturator*" or "nasoalveolar therap*" or dNAM or pNAM).tw,kf. | 7223 |
| 14 | ("presurgical infant orthop?edics" or PSIO or "preoperative orthop?edics" or "pre-operative orthop?edics").tw,kf. | 160 |
| 15 | Orthopedics/ | 27583 |
| 16 | Preoperative Care/ | 49334 |
| 17 | 1 or 2 or 3 | 41687 |
| 18 | 4 or 5 or 6 or 7 or 8 or 9 or 10 or 11 or 12 | 4542071 |
| 19 | 15 and 16 | 150 |
| 20 | 13 or 14 or 19 | 7477 |
| 21 | 17 and 18 and 20 | 92 |

**Cochrane Library**

ID Search Hits

#1 MeSH descriptor: [Cleft Lip] this term only 288

#2 MeSH descriptor: [Cleft Palate] explode all trees 350

#3 cleft near/2 (lip or palate) 1180

#4 #1 OR #2 OR #3 1180

#5 MeSH descriptor: [Computer-Aided Design] this term only 252

#6 "computer aided" near/2 (design or manufacturing) 560

#7 "computer assisted" near/2 (design or manufacturing) 118

#8 "CAD-CAM" or "CAD/CAM" 494

#9 MeSH descriptor: [Printing, Three-Dimensional] this term only 113

#10 (three dimension* or "3 dimension*" or 3D or "3-D") near/2 print* 832

#11 reverse near/2 engin* 24

#12 model* 163922

#13 ((digital or virtual) near/2 (workflow or design or techniqu* or method*)) 1003

#14 #5 OR #6 OR #7 OR #8 OR #9 OR #10 OR #11 OR #12 OR #13 165840

#15 "nasoalveol* molding" or NAM or "palatal obturator*" or "nasoalveolar therap*" or dNAM or pNAM 3337

#16 "presurgical infant orthopedics" or "presurgical infant orthopaedics" or "PSIO" 4

#17 "preoperative orthopedics" 1

#18 "pre-operative orthopedics" 0

#19 "pre-operative orthopaedics" 0

#20 #15 OR #16 OR #17 OR #18 OR #19 3342

#21 #4 AND #14 AND #20 27

**Scopus**

( TITLE-ABS-KEY ( "cleft lip"  OR  "cleft palate" )  AND  TITLE-ABS-KEY ( "computer aided design"  OR  "computer aided manufacturing"  OR  "computer assisted design"  OR  "computer assisted manufacturing"  OR  "CAD/CAM"  OR  "CAD-CAM"  OR  "three dimensional printing"  OR  "3 dimensional printing"  OR  "3D printing"  OR  "3-D printing"  OR  "reverse engineer*"  OR  model*  OR  "digital workflow*"  OR  "digital design"  OR  "digital technique*"  OR  "digital method*"  OR  "virtual workflow*"  OR  "virtual design"  OR  "virtual technique*"  OR  "virtual method*" )  AND  TITLE-ABS-KEY ( "nasoalveol* molding"  OR  nam  OR  "palatal obturator*"  OR  "nasoalveolar therap*"  OR  dnam  OR  pnam  OR  "presurgical infant orthopedics"  OR  "presurgical infant orthopaedics"  OR  psio  OR  "preoperative orthopedics"  OR  "preoperative orthopaedics"  OR  "pre-operative orthopedics"  OR  "pre-operative orthopaedics" ) )

**Web of Science**

ALL=("cleft lip" or "cleft palate") AND ALL=("computer aided design" or "computer aided manufacturing" or "computer assisted design" or "computer assisted manufacturing" or "CAD/CAM" or "CAD-CAM" or "three dimensional printing" or "3 dimensional printing" or "3D printing" or "3-D printing" or "reverse engineer*" or model* or "digital workflow*" or "digital design" or "digital technique*" or "digital method*" or "virtual workflow*" or "virtual design" or "virtual technique*" or "virtual method*") AND ALL=("nasoalveol* molding" or NAM or "palatal obturator*" or "nasoalveolar therap*" or dNAM or pNAM or "presurgical infant orthopedics" or "presurgical infant orthopaedics" or PSIO or "preoperative orthopedics" or "preoperative orthopaedics" or "pre-operative orthopedics" or "pre-operative orthopaedics")

**2. Screening Questionnaires**

Level 1 Screening Questionnaire used to scan the titles and abstracts of articles.

| **Screening Questions** | **Yes**  Include | **No**  Exclude |
| --- | --- | --- |
| **Language:** Is this study written in English? |  |  |
| **Publication Type:** Is this study a case series, case control study, pilot study, randomized/non-randomized controlled trial, or cohort study? |  |  |
| **Population:** Does this study include infants with unrepaired, non-syndromic, complete UCLP or BCLP? |  |  |
| **Discipline:** Does this study examine presurgical infant orthopedics? |  |  |
| **Topic:** Does this study focus on CAD/CAM technology for conducting NAM? |  |  |

Level 2 Screening Questionnaire used to review the full texts of articles to determine eligibility.

| **Screening Questions** | **Yes**  Include | **No**  Exclude |
| --- | --- | --- |
| **Language:** Is this study written in English? |  |  |
| **Publication Type:** Is this study a case series, case control study, pilot study, randomized/non-randomized controlled trial, or cohort study? |  |  |
| **Population:** Does this study include infants with unrepaired, non-syndromic, complete UCLP or BCLP? |  |  |
| **Discipline:** Does this study examine presurgical infant orthopedics? |  |  |
| **Topic:** Does this study focus on CAD/CAM technology for conducting NAM? |  |  |
| **Interventions:** Does this study report on their data acquisition method (e.g. direct intra-oral scanning versus cast scanning)? |  |  |
| **Interventions:** Does this study describe methods for conducting digital molding? |  |  |
| **Interventions:** Does this study describe methods for manufacturing NAM plates? |  |  |
| **Outcome:** Do the results demonstrate that CAD/CAM NAM improved clinical outcomes? |  |  |
| **Decision:** Does this study satisfy all screening criteria, and at least 1 category of the interventions? |  |  |
